# Probiotics Can Generate FoxP3 T-Cell Responses in the Small Intestine and Simultaneously Inducing CD4 and CD8 T Cell Activation in the Large Intestine

**DOI:** 10.1371/journal.pone.0068952

**Published:** 2013-07-04

**Authors:** Maaike J. Smelt, Bart J. de Haan, Peter A. Bron, Iris van Swam, Marjolein Meijerink, Jerry M. Wells, Marijke M. Faas, Paul de Vos

**Affiliations:** 1 Top Institute Food and Nutrition, Wageningen, The Netherlands; 2 Section Immunoendocrinology, Department of Pathology and Medical Biology, University Medical Center Groningen, University of Groningen, Groningen, The Netherlands; 3 NIZO Food Research, Ede, The Netherlands; 4 Host-Microbe Interactomics, Wageningen University, Wageningen, The Netherlands; Charité, Campus Benjamin Franklin, Germany

## Abstract

Most studies on probiotics aim to restore intestinal homeostasis to reduce immune-pathology in disease. Of equal importance are studies on how probiotics might prevent or delay disease in healthy individuals. However, knowledge on mechanisms of probiotic actions in healthy individuals is scarce. To gain more insight in how different bacterial strains may modulate the healthy intestinal immune system, we investigated the effect of the food derived bacterial strains *L. plantarum* WCFS1, *L. salivarius* UCC118, and *L. lactis* MG1363, on the intestinal regulatory immune phenotype in healthy mice. All three bacterial strains induced an upregulation of activity and numbers of CD11c^+^ MHCII^+^ DCs in the immune-sampling Peyer’s Patches. Only *L. salivarius* UCC118 skewed towards an immune regulatory phenotype in the small intestinal lamina propria (SILP). The effects were different in the large intestine lamina propria. *L. salivarius* UCC118 induced activation in both CD4 and CD8 positive T-cells while *L. plantarum* WCFS1 induced a more regulatory phenotype. Moreover, *L. plantarum* WCFS1 decreased the Th1/Th2 ratio in the SILP. Also *L. lactis* MG1363 had immunomodulatory effects. *L. lactis* MG1363 decreased the expression of the GATA-3 and T-bet in the SILP. As our data show that contradictory effects may occur in different parts of the gut, it is recommended to study effects of probiotic in different sites in the intestine. Our strain-specific results suggest that unspecified application of probiotics may not be very effective. Our data also indicate that selection of specific probiotic strain activities on the basis of responses in healthy mice may be a promising strategy to specifically stimulate or suppress immunity in specific parts of the intestine.

## Introduction

Intestinal microorganisms are of essential importance for the development and maintenance of homeostasis of both the intestinal and peripheral immune system [1], [2]. This is illustrated by the fact that an altered microbiota is associated with the development of intestinal infections and inflammatory bowel disease (IBD) [3], [4]. Modification of the intestinal microbiota by administration of probiotic bacteria, such as *Lactobacillus* or *Bifidobacterium* species, is a promising strategy to prevent or overcome excessive intestinal inflammation and to restore immune homeostasis [5]–[9]. The efficacy of probiotics in the treatment of intestinal inflammation has been demonstrated in a range of experimental disease models [10]–[12], as well as in patients suffering from intestinal inflammatory diseases [13]–[17].

Besides the beneficial effects of probiotics in inflammatory disease, the disease-preventing potential of probiotic bacteria is gaining attention [18]–[21]. Probiotic treatment may benefit individuals who are not yet receiving medical treatment, but are at risk of developing disease due to age- [22], obesity- [23], malnutrition- [24], or stress-related [25] deterioration of immune homeostasis. Surprisingly, the number of studies describing the immunomodulatory effects of probiotic bacteria in non-diseased individuals is small [18]–[21]. Most studies have focused on the diseased situation to demonstrate the efficacy of probiotic treatment [5]–[9], [13]–[17]. However, due to immune pathology [26], [27] and a disruption of the intestinal barrier [28], these studies may not reflect or predict the immunomodulatory effects of probiotics in healthy individuals or persons with sub-optimal immune health. Studying how different bacterial strains influence the immune system in the healthy intestine will provide insight in the mechanisms of beneficial effects of probiotics in the intestine. Further, it will provide insight in the strain dependency of probiotic treatment, their safety, as well as potential applications for improving or maintaining immune health. For these reasons, we decided to investigate the immunomodulatory effects of probiotic bacteria in healthy, non-diseased mice.

It is hypothesized that in the intestine, probiotics influence the intestinal immune response through several different pathways: i) probiotic sampling in the Peyer’s Patches (PP), influencing dendritic cell (DC) and T cell responses in and beyond the PP; (ii) probiotic-interaction with small intestine lamina propria (LP) DCs, of which at least two specialized subsets can be discriminated, CD103^+^CX_3_CR1^−^ DCs and CD103^−^CX_3_CR1^+^ DCs [29], influencing LP T cell responses; and (iii) probiotic-interaction with epithelial cells, influencing DC and T cell responses in the LP through the secretion of cytokines [30], [31]. Although most of the research on probiotic-induced immune signaling has focused on the small intestine [32], also the large intestinal LP is an immunological effector site [33] in which changes are associated with the development of inflammatory bowel diseases, such as ulcerative colitis.

In the present study we investigated the distribution of DC and T cell subsets at different intestinal induction and effector sites [peyers patches (PP), small intestinal LP (SILP) and in the large intestinal LP (LILP)] after administration of *L. plantarum* WCFS1 [21], *L. salivarius* UCC118 [20], and *L. lactis* MG1363 [34] to healthy mice. The bacteria were administered over 5 days, covering the period required for mice to develop an adaptive immune response [18], [35]. In this study, we demonstrated strain-dependent effects of the bacterial treatments on DC and T cell activation in the PP, SILP and LILP, as well as reduction of the Th1/Th2 ratio in the small intestinal LP. We demonstrated that *L. salivarius* UCC118 and *L. plantarum* WCFS1 have the strongest immunomodulating capacities of the three tested bacterial strains. *L. salivarius* UCC118 skews the balance between effector and regulatory T cells towards an immune regulatory phenotype in the small intestinal LP while simultaneously activating T-cells in the large intestinal LP. *L. plantarum* WCFS1 skews the balance towards an immunoregulatory phenotype in the large intestinal LP, while at the same time this probiotic shifted the Th1/Th2 balance towards Th2 in the small intestine LP. This warrants caution in drawing conclusions about the type of immunomodulating capacity of a specific strain when only one location in the intestine is studied. Although less pronounced, also *L. lactis* MG1363 had immunomodulating effects.

## Materials and Methods

### Bacterial Strains and Growth Conditions

*L. plantarum* WCFS1 [36] and *L. salivarius* UCC118 [37] were cultured at 37°C in Man, Rogosa, and Sharpe (MRS) broth. *L. lactis* MG1363 [38] was cultured at 30°C in M17 broth containing 0.5% glucose. All bacterial cultures were grown overnight until the stationary phase of growth was reached. Subsequently, the cultures were diluted 1∶1000 in fresh medium and cultured for a second night. The optical density at 600 nm was measured and the number of colony forming units (CFU) was calculated based on standard growth curves. For all cultured bacterial strains, an OD_600_-value of 1 corresponds to 1–2×10^9^ CFU/mL, which was confirmed by plating serial dilutions on MRS or M17 agar plates. To avoid bacterial alteration and cell death, extensive washing and centrifugation was avoided. After overnight growth, bacteria were diluted in fresh, sterile MRS and immediately administrated to the animals. The mice received either sterile MRS as a control or 1–2×10^8^ CFU bacteria in 200 µL MRS via intragastric gavage, daily.

### Animals and Tissue

Wild-type male Balb/c mice were purchased from Harlan (Harlan, Horst, The Netherlands). The animals were fed standard chow and water *ad libitum*. All animal experiments were performed after receiving approval of the institutional Animal Care Committee of the Groningen University (DEC5644B). All animals received animal care in compliance with the Dutch law on Experimental Animal Care.

To study the effect of three bacterial strains on the systemic immune system (*L. lactis* MG1363, *L. salivarius* UCC118, and *L. plantarum* WCFS1) or that of MRS broth, a 200 µl volume sample was administered by intragastric gavage of once daily for five consecutive days [18]. At day six, the mice were sacrificed, after which the intestine was removed for further analysis. A tissue sample from the middle of the small intestine (i.e. ileum) was snap frozen in liquid nitrogen for RNA isolation and quantitative real-time PCR. Due to restrictions in the cellular yield, only the PP, SILP and the large intestine were used for cell isolations. The LILP cell yield was not high enough to allow accurate analyses of (activated) (CD103^+^) dendritic cell frequencies. Therefore, only T cell subsets were analyzed in the LILP.

### Cell Isolation

After sacrificing the mice, the intestine was removed and rinsed with ice cold PBS. PPs were removed from the tissue and single cell suspensions were made by mechanical disruption of the tissue between two glass slides in 1 mL of ice cold RPMI containing 10% heat inactivated fetal calf serum (FCS). Subsequently, a cell strainer was used to remove remaining cell clumps.

The small and large intestine were cut in small pieces and rinsed three times in ice cold Phosphate Buffered Saline (PBS). Epithelial cells were removed by incubation of the tissue in PBS containing 10% heat inactivated FCS, 1 mM Sodium Pyruvate, 10 mM Ethylenediaminetetraacetic (EDTA) and 20 mM 4-(2-hydroxyethyl)-1-piperazine-ethanesulfonic acid (HEPES) (pH 7.4) for 30 minutes at 37°C. Subsequently, the tissue was washed in ice cold PBS. The LP was removed by incubation of the tissue in RPMI 1640 medium, containing 10% heat inactivated FCS, 1.5 mg/mL Collagenase D (Sigma Aldrich), and 10 mg/mL DNAse I (Sigma Aldrich), for 60 minutes at 37°C. The reaction was terminated by the addition of EDTA to a final concentration of 10 mM. The cell suspension was washed in ice cold PBS. A cell strainer was used to remove remaining cell clumps.

Lymphocytes were enriched and dead cells were removed from the PP and LP mixtures by resuspension in 20% percoll and loading on a 55%, 45%, and 35% percoll gradient (GE Healthcare, Eindhoven, the Netherlands). Gradients were centrifuged at room temperature for 30 minutes at 800 *g.* The interface containing live cells was collected and washed in ice cold PBS, before cell counting and staining. After density gradient centrifugation, more than 90% of the cells were vital, which was confirmed by propidium iodide staining.

### Cell Staining

T cell stainings were performed on single cell suspensions retrieved from the Peyer’s patches (PP), small intestinal LP (SILP), and large intestinal LP (LILP). DC stainings were performed on cells retrieved from the PP and SILP. The T cell cocktail contained monoclonal antibodies directed against CD3, CD4, CD8, CD25, CD69, FoxP3, or appropriate isotype controls (Table 1). The DC cocktail contained monoclonal antibodies directed against CD11c, MHC II, CD19, CD80, CD86, CD103, or appropriate isotype controls (Table 1).

**Table 1:** Antibodies used for flow cytometry.

Specificity	CloneName	Fluorochrome	Dilution	Supplier
CD3	17A2	Pacific Blue	200x	BioLegend
CD4	RM4–5	PerCP	200x	BioLegend
CD8	53-6.7	Alexa700	50x	BioLegend
CD25	3C7	APC	100x	BioLegend
CD69	H1.2F3	PE	200x	BioLegend
FoxP3	FJK-16S	FITC	100x	eBioscience
Rat IgG2b	N/A	APC	100x	BioLegend
Hamster IgG	N/A	PE	200x	BioLegend
Rat IgG2a	N/A	FITC	100x	eBioscience
CD11c	N418	APC	25x	BD Biosciences
MHC II	2G9	Biotin +streptavidin PerCP	150x	BD Biosciences
CD19	6D5	PE-Cy7	100x	BioLegend
CD80	16-10A1	PE	50x	BioLegend
CD86	PO3	Alexa700	50x	BioLegend
CD103	2E7	Pacific Blue	25x	BioLegend
Hamster IgG	N/A	PE	50x	BioLegend
Rat IgG2b	N/A	Alexa700	50x	BioLegend
Hamster IgG	N/A	Pacific Blue	25x	BioLegend

Briefly, 1×10^6^ cells were incubated in FACS buffer (PBS containing 2% heat-inactivated FCS) containing 10% normal mouse serum for 30 minutes to prevent non-specific antibody staining. Subsequently, the cells were incubated with a cocktail of primary antibodies for 30 minutes, in the dark, after which the cells were washed in ice cold FACS buffer twice. Tubes stained for T cells were subsequently fixed in ice cold 1×FACS Lysing solution (BD Biosciences) for 30 minutes in the dark and washed twice in permeabilisation buffer (eBioscience). Subsequently, the cells were incubated with the anti-FoxP3 antibody or the isotype in permeabilisation buffer containing 2% rat serum for 30 minutes in the dark. Then the cells were washed twice in ice cold permeabilisation buffer and resuspended in FACS buffer until FACS analysis. Tubes for DC staining were washed twice in ice-cold FACS buffer, after which the cells were incubated with the secondary step for 30 minutes in the dark. Subsequently, the cells were fixed in ice cold FACS Lysing solution for 30 minutes in the dark followed by washing twice in ice cold FACS buffer and resuspended in FACS buffer until FACS analysis (within 24 hours). The whole procedure was performed on ice.

### Flow Cytometry

At least 5×10^5^ cells were analyzed by flow cytometry. Flow cytometry was performed using the LSR II Flow Cytometer system (BD Pharmingen), with FACS Diva software. Analysis was performed using FlowJo 7.6.2 software.

**Figure 1:**
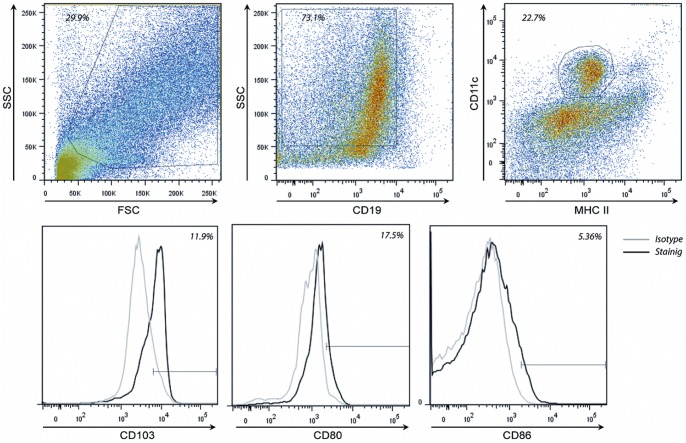
Dendritic cells were gated in the forward side scatter plot, based on size and granularity. CD19^+^ B-cells were excluded from analysis and the frequency of MHC II^+^ CD11c^+^ cells was determined. Within the DC populations CD80, CD86, or CD103 isotype controls were used to set the gate to 99% negative cells. This gate was copied to the samples stained for CD80, CD86, and CD103 and the frequency of positive cells was determined.

Dendritic cells were gated in the forward side scatter plot, based on size and granularity, CD19^+^ B-cells were excluded from analysis and the frequency of MHC II^+^ CD11c^+^ cells was determined (Figure 1). Within the DC populations CD80, CD86, or CD103 isotype controls were used to set the gate to 99% negative cells. This gate was copied to the sample stained for CD80, CD86, and CD103 and the frequency of positive cells was determined (Figure 1). DCs were defined as CD11c^+^MHC II^+^ cells. Intestinal DCs are depicted as the frequency of CD103^+^ cells within the CD11c^+^MHC II^+^ population. CX_3_CR_1_^+^ DCs were defined as CD11c^+^ MHC II^+^ CD103^−^ DCs. Also the frequency of activated DCs was determined and expressed as the frequency of CD80^+^ or CD86^+^ cells within the CD11c^+^MHC II^+^ cell population.

Lymphocytes were gated in the forward side scatter plot and the frequency of CD3^+^ T cells was determined. Within the T cell population the frequency of CD8^+^ T cells and CD4^+^ T cells was determined. Within both the CD4 and CD8 T cell population the isotype controls for CD69 or CD25 were used to set the gate to 99% negative cells. This gate was then copied to the sample stained for CD69 or CD25 and the frequency of positive cells was determined.

Regulatory T cells are defined based on the expression of CD25 and the transcription factor FoxP3. For this, FoxP3^+^ cells were gated within the CD4 T cell population (within the CD4 T cell population the FoxP3 isotype control was used to set the gate to 99% negative cells). It has been shown by us and others that these cells express regulatory cytokines [39], [40]. This gate was copied to the sample stained for FoxP3 and the frequency of positive cells was determined. The expression of CD25 in these cells was confirmed. All CD4^+^FoxP3^+^ cells consistently demonstrated CD25 expression. Results are expressed as the frequency of CD25^+^FoxP3^+^ cells within the total CD4 T cell population (CD4^+^CD3^+^ cells). Effector T cells are defined as the frequency of CD25^+^FoxP3^−^ or CD69^+^ cells within the CD4^+^CD3^+^ (T helper cells) or CD8^+^CD3^+^ (cytotoxic T cells; CTLs) population.

### qRT-PCR

mRNA of immunological genes was detected by quantitative reverse transcribed PCR (qRT-PCR). The tissues (*N = 6* per group) were lyzed in Trizol lysing buffer (Invitrogen). Total RNA was extracted by chloroform-isopropanol extraction. cDNA was prepared from isolated RNA using Superscript™ II Reverse Transcriptase according to the kit protocol (Invitrogen). Quantitative real time RT-PCR was performed on the ABI7900 Taqman (Applied Biosystems) using a two-step amplification protocol. PCR reactions contained 10 ng/mL of cDNA as template, 1.5 µM forward and reverse primer and SYBR Green PCR master mix (Applied Biosystems) in a total reaction volume of 20 µL. All PCR reactions were performed in triplicate. Relative gene expression was normalized to the GAPDH expression (ΔCt = Ct_GENE OF INTEREST_- Ct_GAPDH_) of the same sample and depicted as inverted relative expression levels [1/ΔCt (A.U.)]. Primer sequences are described in Table 2. All PCR reactions were optimized using RNA isolated from the spleens of untreated Balb/c mice.

**Table 2:** Primer sequences used for quantitative real-time PCR.

Transcript	Forward Primer	Reverse Primer
**T-bet**	GCCAGGGAACCGCTTATATG	GACGATCATCTGGGTCACATTGT
**GATA-3**	AGGCAAGATGAGAAAGAGTGCCTC	CTCGACTTACATCCGAACCCGGTA
**RORγT**	CACGGCCCTGGTTCTCAT	CAGATGTTCCACTCTCCTCTTCTCT
**IL4**	ACAGGAGAAGGGACGCCAT	GAAGCCCTACAGACGAGCTCA
**IL5**	AGCACAGTGGTGAAAGAGACCTT	TCCAATGCATAGCTGGTGATTT
**IL10**	GGTTGCCAAGCCTTATCGGA	ACCTGCTCCACTGCCTTGCT
**IL12p40**	GGAAGCACGGCAGCAGAATA	AACTTGAGGGAGAAGTAGGAATGG
**IL17**	ATCAGGACGCGCAAACATGA	TTGGACACGCTGAGCTTTGA
**IL23p19**	TGTGCCCCGTATCCAGTGT	CGGATCCTTTGCAAGCAGAA
**IFNγ**	TCCTGCAGAGCCAGATTATCTC	CTCGGATGAGCTCATTGAATGC
**TGFβ**	GGGCTACCATGCCAACTTCTG	GAGGGCAAGGACCTTGCTGTA

### Statistics

Flow cytometry data results are expressed as the mean ± standard error of the mean (SEM). Normal distribution of the data sets was confirmed by the Kolmogorov-Smirnov test. The two-sided Students t-test was used to determine changes in immune cell populations after probiotic treatment. Gene expression data are expressed as the median (range). The Th1/Th2 ratio was evaluated by dividing the gene expression of T-bet by gene expression of GATA-3. The two-sided Mann Whitney U-test was used to determine changes in expression profiles after probiotic treatment *in vivo.* P-values <0.05 (*) were considered statistically significant.

## Results

### Probiotic Treatment Induces DC and T cell Activation in the Small Intestinal PP

We evaluated the immunomodulatory properties of *L. plantarum* WCFS1, *L. salivarius* UCC118, and *L. lactis* MG1363 *in vivo.* These strains were selected for their high IL10 inducing potential in murine bone marrow derived dendritic cells as shown in a previous study from our group [18]. We first focused on the Peyer’s Patches (PP), the mucosal sites for induction of adaptive immune responses [41]. The mice (*N = 6* per group) received the bacteria, or culture medium alone as a control, for 5 consecutive days.

**Figure 2:**
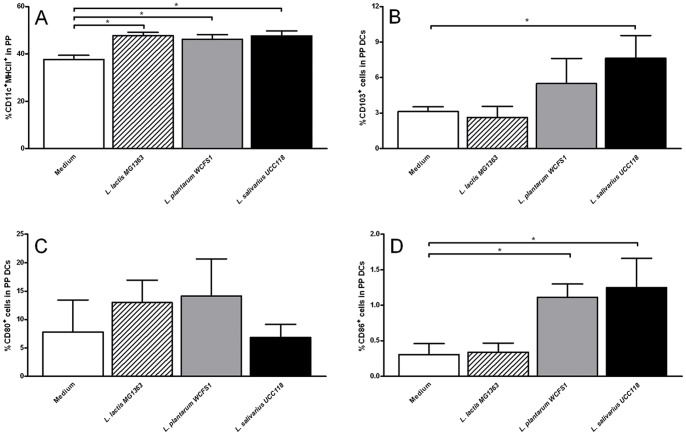
Effects of three bacterial strains on Peyer’s Patch dendritic cells. Frequency of CD11c+ MHC II+ dendritic cells (A), CD103+ dendritic cells (B), CD80+ dendritic cells (C), or CD86+ dendritic cells (D) in the Peyer’s Patches following treatment with culture medium (white bars) (N = 6), *L. lactis* MG1363 (dashed bars) (N = 6), *L. plantarum* WCFS1 (grey bars) (N = 6), or *L. salivarius* UCC118 (black bars) (N = 6). CD103, CD80, and CD86 frequencies are expressed as the frequency of cells within the CD11c+ MHC II+ population. Results are expressed as the mean ± standard error of the mean (SEM). Statistical significance was calculated using the Students t- test. * represents P-values <0.05.

**Figure 3:**
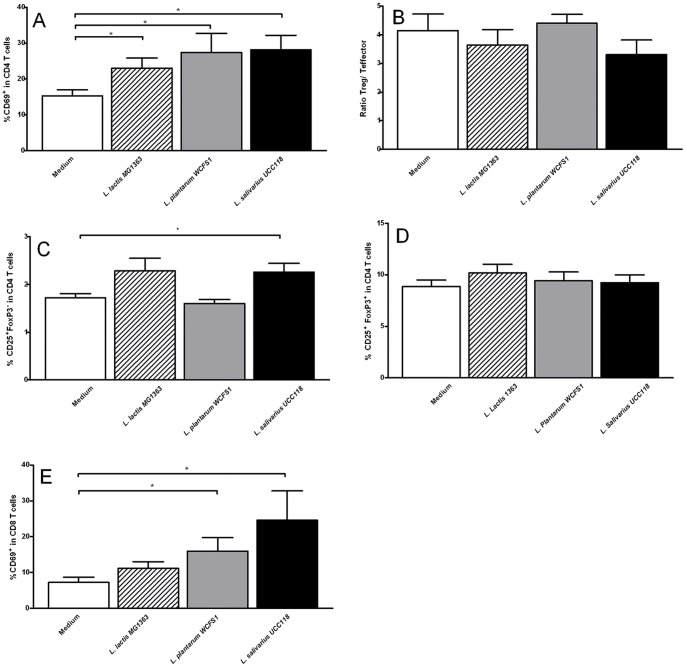
Effects of three bacterial strains on Peyer’s Patch T cells. Frequency of CD69+ CD4 T cells (A), regulatory T/effector T cell ratio (B), CD25+ FoxP3- effector CD4 T cells (C), CD25+ FoxP3+ regulatory T cells (D), or CD69+ CD8 T cells (E) in the Peyer’s Patches following treatment with culture medium (white bars) (N = 6), *L. lactis* MG1363 (dashed bars) (N = 6), *L. plantarum* WCFS1 (grey bars) (N = 6), or *L. salivarius* UCC118 (black bars) (N = 6). Results are expressed as the mean ± standard error of the mean (SEM). Statistical significance was calculated using the Students t-test. * represents P-values <0.05.

In the PP, the first immune cells to respond to transcytosed antigens are the dendritic cells in the dome area, underlying the follicular epithelium [41]. All bacteria-treated groups demonstrated increased CD11c^+^ MHC II^+^ dendritic cell frequencies in the PP as compared to the medium treated mice (Figure 2A). The percentage of CD103^+^ intestinal DCs was increased, while the percentage of CD103- intestinal DCs (i.e. CX_3_CR_1_^+^ DCs; results not shown) was decreased, but both only reached statistical significance after *L. salivarius* UCC118 administration (Figure 2B). Although no effect of probiotic treatment was observed on % CD80+ DCs in the PP (Figure 2C), the treatment with *L. plantarum* WCFS1 and *L. salivarius* UCC118, but not *L. lactis* MG1363 did increase the activation status of the dendritic cells in the PP as demonstrated by increased frequencies of CD86^+^ DCs (Figure 2D). These changes in the DC compartment of the PP coincided with a twofold increase in early activated CD4 T cells, as demonstrated by increased CD69^+^ CD4 T cell frequencies following *L. plantarum* WCFS and *L. salivarius* UCC118 (fig 3A) and *L. lactis* MG1363 treatment (Figure 3A) and increased percentage of CD25+ cells after *L. salivarius* UCC118 treatment as compared with medium treatment (Figure 3C). The balance between effector CD4 T cells and regulatory T cells in the PP (Figure 3B and 3D) was not changed. The frequency of early-activated CD69^+^ CD4 T cells in the CD8+ compartment was strongly increased by *L. plantarum* WCFS and *L. salivarius* UCC118 treatment but not by *L. lactis* MG1363 treatment (Figure 3E).

### *L. salivarius* UCC118 but not *L. plantarum* WCFS or *L. lactis* MG1363 Skews T cells Towards a Regulatory Phenotype in the Small Intestinal LP

Next, we questioned whether the intestinal effector sites are altered by the bacterial treatments. For this, we studied the distribution of immune cell populations in the small intestinal LP (SILP). In addition to analysis of DC and T cell subsets by flow cytometry, the expression levels of T cell polarizing cytokines were determined by quantitative real-time RT-PCR as a measure for specific T effector cell responses.

**Figure 4:**
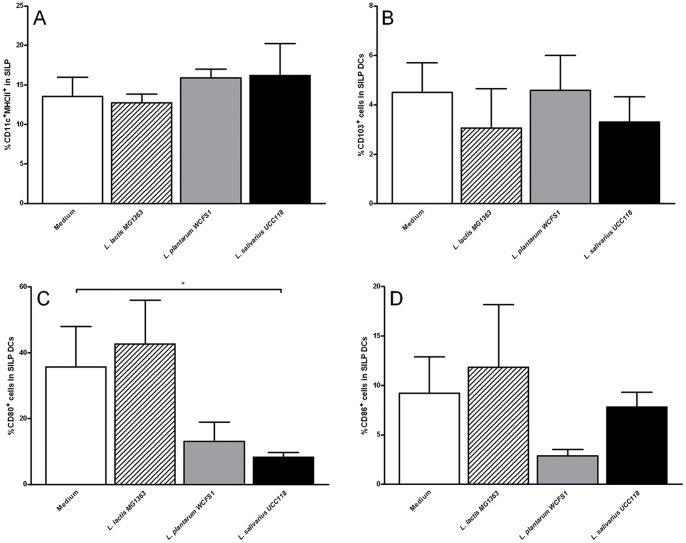
Effects of administration of three types of bacterial strains on the small intestinal LP (SILP) dendritic cells. Frequency of CD11c+ MHC II+ dendritic cells (A), CD103+ dendritic cells (B), CD80+ dendritic cells (C), or CD86+ dendritic cells (D) in the SILP following treatment with culture medium (white bars) (N = 6), *L. lactis* MG1363 (dashed bars) (N = 6), *L. plantarum* WCFS1 (grey bars) (N = 6), or *L. salivarius* UCC118 (black bars) (N = 6). CD103, CD80, and CD86 frequencies are expressed as the frequency of cells within the CD11c+ MHC II+ population. Results are expressed as the mean ± standard error of the mean (SEM). Statistical significance was calculated using the Students t- test. * represents P-values <0.05.

None of the bacterial treatments significantly altered the frequency of total DCs (Figure 4A), CD103^+^ (Figure 4B) or CD103^−^ (not shown) DCs in the SILP. We did observe however a strong reduction of the CD80+ dendritic cells in the SILP after *L. plantarum* WCFS and *L. salivarius* UCC118 treatment. This however only reached statistical significant differences with *L. salivarius* UCC118 (Figure 4C) in the SILP. These effects were less pronounced on the CD86+ expressing DC compartment (Figure 4D).

**Figure 5:**
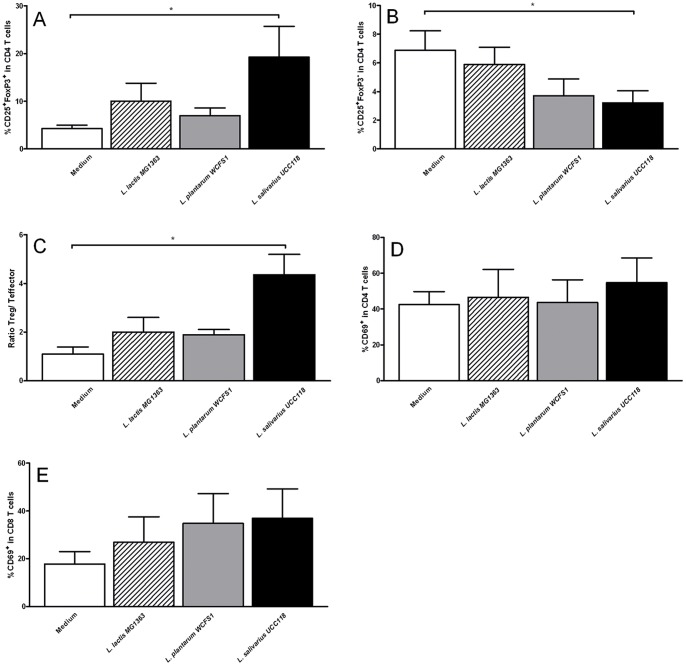
Effects of administration of three types of bacterial strains on the small intestinal LP (SILP) T cells. Ratio of regulatory T/effector T cells (A), frequency of CD25+ FoxP3- effector CD4 T cells (B), CD25+ FoxP3+ regulatory T cells (C), CD69+ CD4 T cells (D), CD69+ CD8 T cells (E) in the SILP following treatment with culture medium (white bars) (N = 6), *L. lactis* MG1363 (dashed bars) (N = 6), *L. plantarum* WCFS1 (grey bars) (N = 6), or *L. salivarius* UCC118 (black bars) (N = 6). Results are expressed as the mean ± standard error of the mean (SEM). Statistical significance was calculated using the Students t- test. * represents P-values <0.05.

*L. salivarius* UCC118 treatments skewed the balance between effector and regulatory T cells to a more regulatory phenotype (Figure 5C). In the *L salivarius* UCC118 treated animals, this altered balance was caused by a pronounced decrease of effector T cell frequencies (Figure 5B), combined with an increased regulatory T cell frequencies (Figure 5A).

The frequency of activated CD69^+^ CD4 T cells and activated CD8 T cells (Figure 5D, 5E) increased in most cases but was variable and did, therefore, not reach statistical significant differences.

### *L. lactis* MG1363 and *L. plantarum* WCFS1 Treatment Decreases Th1 and Th2 Specific Cytokine Expression in the Small Intestinal LP

By PCR we quantified the expression of T-bet, GATA-3, RORγT, IL4, IL5, IL10, IL12p40, IL17, IL23p19, IFNγ, TGFβ in the SILP. We found only statistical significant changes in T cell polarization transcription factors. The expression of the Th2-specific transcription factor GATA-3 was significantly decreased in response to *L. lactis* MG1363 and *L. plantarum* WCFS1 treatment (P<0.05) (Table 3). Although, these 2 bacterial strains also decreased the expression of the Th1 transcription factor T-bet (P<0.05) (Table 3), the resulting Th1/Th2 ratio was decreased in both cases, illustrating a more pronounced skewing towards Th2. This Th1/Th2 ratio decrease only reached statistical significance after treatment with *L plantarum* WCFS1.

**Table 3:** Gene expression levels in the small intestine lamina propria.

Transcript	Medium	*L. lactis* MG1363	*L. plantarum* WCFS1	*L. salivarius* UCC118
**T-bet**	0.093713±0.007254	0.045034±0.016484*	0.026414±0.017406*	0.058556±0.025691
**GATA3**		0.08584±0.01587	0.035086±0.018961*	0.032161±0.016688*
**Th1/Th2 ratio**	1.244727±0.226619	1.189644±0.555112	0.396565±0.25265*	0.864353±0.333015
**RORγT**	0.173445±0.028785	0.185399±0.018323	0.184259±0.006646	0.209058±0.036537
**TGFβ**	0.130745±0.05392	0.136491±0.024243	0.131613±0.007886	0.198002±0.068527

Of all primer sequences shown in Table 2, only T-bet and GATA-3, RORγT and TGBβ were detected and are shown in this table. By dividing T-bet through GATA-3 expression the Th1/Th2 ratio is calculated.

* represents P-values <0.05.

Th17 transcription factor RORγT transcripts were abundantly present in the SILP, but the expression levels were not changed by the probiotic treatments (Table 3). Similarly, IL17 expression was not influenced by probiotic treatment (data not shown). Expression of the Th1 cytokines IL12p40 and IFNγ was low. The regulatory IL10 and FoxP3 transcripts were low and not altered by the treatments (data not shown). TGFβ, which is involved in both T cell skewing towards a regulatory phenotype as well as skewing towards a Th17 phenotype [42], was abundantly present in the SILP (Table 3) but not altered by the probiotic treatments. The expression levels of the cytokines IL-4 and IL-5 were not detected or in very low levels and were not affected by probiotic treatment (results not shown).

### Probiotic Treatment Induces T cell Activation in the Large Intestinal LP

Although the mucosal interface of the large intestine is much smaller than that of the small intestine, the LP of the large intestine (LILP) is also considered an immune effector site. On average, 746,000±7875 cells were retrieved from the LILP, which was too low to allow for reliable quantification of changes in the small DC compartment. Therefore, only changes in the T cell compartment were analysed.

**Figure 6:**
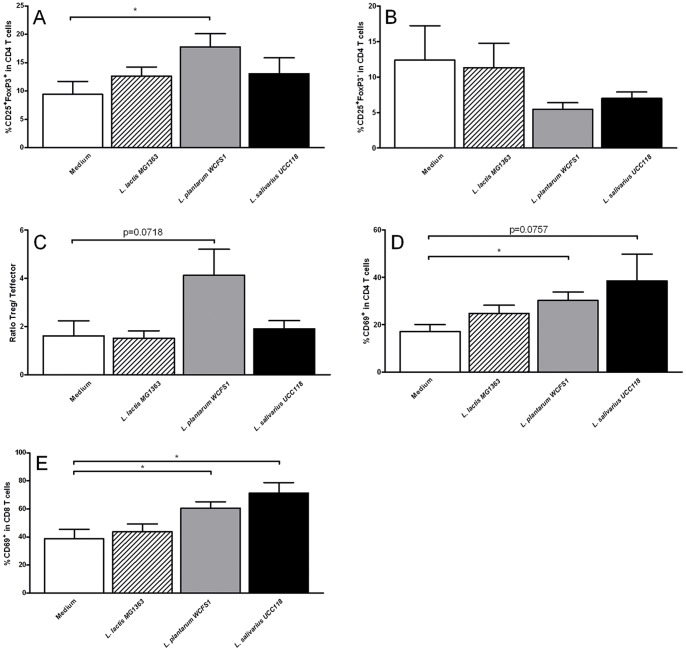
Effects of administration of three types of bacterial strains on the large intestinal LP (LILP) T cells. Frequency of CD25+ FoxP3+ regulatory T cells (A), CD25+ FoxP3- effector CD4 T cells (B), ratio of regulatory T/effector T cells (C), CD69+ CD4 T cells (D), or CD69+ CD8 T cells (E) in the LILP following treatment with culture medium (white bars) (N = 6), *L. lactis* MG1363 (dashed bars) (N = 6), *L. plantarum* WCFS1 (grey bars) (N = 6), or *L. salivarius* UCC118 (black bars) (N = 6). Results are expressed as the mean ± standard error of the mean (SEM). Statistical significance was calculated using the Students t- test. * represents P-values <0.05.

The effects were strain dependent. The frequency of regulatory T cells was significantly enhanced by *L. plantarum* WCFS1 treatment (Figure 6A). As a consequence the regulatory-effector balance after *L. plantarum* WCFS1 treatment showed a trend towards a more regulatory environment in the LILP (*p = 0.07*) (Figure 6C). However, both *L. plantarum* WCFS1 and *L. salivarius* UCC118 treatment enhanced CD4 and CD8 T cell activation, as demonstrated by increased frequencies of CD69^+^ expressing cells (Figure 6D and 6E respectively). *L. lactis* MG1363 treatment had no effect on LILP CD4 and CD8 T cells (Figure 6).

## Discussion

The majority of probiotics are marketed for consumption by healthy individuals to prevent disease. However, to date most experimental studies have focused on specific intestinal disease models to demonstrate the efficacy of probiotics [10]–[12]. In these models, the intestinal immune barrier may be compromised, altering the contact between the probiotic bacteria and the intestinal immune cells [28], [43], [44]. Moreover, in these models, immune homeostasis is strongly perturbed and playing a role in the pathophysiology of the disease [26], [27]. Although these models provide valuable insight into the efficacy of the probiotic treatment, they do not reflect or predict the immunomodulatory properties in the healthy situation. For this reason, we decided to study the intestinal immunomodulatory effects of different bacterial strains in healthy mice. The chosen strains are food-derived bacteria [20] with confirmed immunomodulating effects in the murine systemic circulation [18], [40] and on *ex vivo* dendritic cells [40], [45]–[47].

To our knowledge, this is the first report on differential location-specific immune changes in the intestine following short-term treatment with probiotics. After administration of the three bacterial species we always observed an upregulation and increased activity of DC’s in the PP illustrating an enhanced activity in this immune sampling site. *L. salivarius* UCC118 treatment skewed the adaptive immune balance towards a regulatory phenotype in the SILP, but not in the LILP (both immune effector sites). This was different with *L. plantarum* WCFS1. *L. plantarum* WCFS1 had almost no effects on regulatory cells in the SILP but shifted the Th1/Th2 balance towards Th2 in the SILP as GATA-3 suppression was less pronounced than T-bet suppression in the SILP. The effects of *L. plantarum* WCFS1 were different in the LILP as here *L. plantarum* WCFS1 induced an upregulation of regulatory cells. This was different with *L. salivarius* UCC118 where in the LILP only enhanced activation of T-cells was observed.

Up to now, we and others explained immunomodulatory effects of probiotics by direct host-probiotic interactions [20], [48]. This explanation was supported by the identification of several effector molecules on probiotics that can interact with pattern recognition receptors found on gut epithelial cells or on intestine-bound immune cells [45]. Although plausible, this theory cannot explain that the same probiotic has a principally different effect in the small and large intestine. This observation does fit however in the theory of the pioneer strategy of probiotic administration. According to this theory a probiotic does not necessarily become a residential part of the host microbiome but may benefit other bacteria in the intestine [49]. As the microbiome composition is different in the small and large intestine also different effects of probiotic administration may be expected with, consequently, different effects on the host immune system. This however should not be interpreted as a suggestion that direct interaction with the host is not involved in immunomodulation. We have shown in previous studies that short-term administration of probiotics, that cannot induce shifts in the composition microbiota, does result in immediate host-responses [20]. However, with longer administration periods such as in the present study location specific changes in microbiota may occur as well.

In a previous study we showed that probiotics might skew the peripheral immune response away from Th2 responses in healthy mice [18], [47]. Our present study showed that locally in the SILP only two of the three strains attenuate Th2 responses. However, this did not results in a higher Th1/Th2 ratio since in case of *L. plantarum* WCFS1 the T-bet expression was more profoundly suppressed resulting in a threefold decrease in Th1/Th2 ratio. As the same strains were being used in our previous study and this study [18] it has to be concluded that local effects on T-cells are not necessarily reflected in the systemic circulation. This should be explained by the fact that as shown in our previous study [18] many processes are activated in the mesenterial lymph nodes and spleen after probiotic induced immune activation.

Also *L. lactis* MG1363 had a decreasing effect on GATA-3 and T-bet, but this did not result in a significant change in the Th1/Th2 ratio. This effect of *L. lactis* MG1363 is surprising as *L. lactis* MG1363 is not generally considered to be a probiotic strain. This should change as in addition to effects on Th1 and Th2 differentiation, it also activated dendritic cells in the PP. The probiotic effects of this strain have also been found in the systemic circulation in previous studies [18], [47].

Sampling of luminal contents may occur through DCs in the PP [50], goblet cells [51], and LP DCs that exert their dendrites into the intestinal lumen [41], [50]. However, all these studies on immune sampling focus on luminal particles and not on full bacteria such as probiotics. It is still largely unknown where probiotics are sampled. Most likely probiotics are sampled in our study in the PP as we found increased frequencies of antigen-presenting DCs and DC activation in the PP and not that outspoken for all strains in the LP. However, subtle changes in the LP (CD103^+^) DC population may be responsible for the previously observed changes in systemic immunity [18]. However, we should again emphasize that this might be different in (experimental) disease models, in which the barrier function is disrupted [28] and direct contact between the probiotics and immune cells is possible [44], [52].

It might be suggested that advanced *in vitro* tools [35], [46], [53]–[56] might have been helpful in understanding and predicting the effects of the strains in the present study. However, we have applied many of those systems in previous studies [18], [40], [46], [56] but we had to conclude that the majority of the systems are poor predictors as the systems focus on the secretion of only one or a few pro- and anti-inflammatory cytokines from PBMCs or DCs as a model for immunomodulation *in vivo* [35], [46], [53]–[56]. As shown in the present study the composition of the gastrointestinal immune system is very complex as a consequence of which contradictory effects can be found in different parts of the intestine. To our best knowledge there are no *in vitro* models available that have been designed to mimic the different parts of the intestine.

The decreased Th1/Th2 ratio after *L. plantarum* WCFS1 treatment in the SILP may suggest that *L. plantarum* WCFS1 may be effective in the prevention or slowing down the development of Th2 skewed intestinal diseases [27] in still healthy individuals. *L. salivarius* UCC118 may be less effective in this respect but might theoretically be beneficial for preventing or slowing down chronic, low grade intestinal immune disorders which require only a modest adjustment in the Th1/Th2 balance, or intestinal food allergies that require enhanced immunosuppression rather than an altered Th1/Th2 balance [57]. However the differential effects in the SILP and LILP warrant caution in suggesting the application of *L. salivarius* UCC118 in disease models. Especially the enhanced inflammatory responses in the LILP may be problematic when the barrier is disturbed. This dualistic effect of *L. salivarius* UCC118 in different parts of the gut may also explain the variable performance of *L. salivarius* UCC118 in disease models [58]–[60].

In summary, in the current study we demonstrated intestinal immunomodulation following short-term oral administration of three bacterial strains in healthy mice. The observation that these effects on the immune system are strain dependent supports the need to select probiotics for specific groups of individuals with different needs instead of generalized application of probiotics for prevention of any type of disorder. Our data suggest that it may be mandatory to select strains and test specific probiotics to prevent allergy (i.e. suppressing Th2 responses) and others for preventing infection in still healthy individuals (i.e. stimulating Th1 responses). This however should be carefully defined as we show that some probiotics may have desired effects in the small intestine while inducing responses of a proinflammatory nature in other parts of the intestine. Although further research is required, our results suggest that the selection of specific probiotic strains on the basis of responses in healthy mice may be a promising strategy to improve health and prevent specific intestinal immunological disorders.
